# The effect of group art therapy on acculturative and academic stress of Chinese graduate students in South Korea

**DOI:** 10.3389/fpsyg.2023.1179778

**Published:** 2023-07-20

**Authors:** Yue Yin, Kyung Soon Ko

**Affiliations:** ^1^Kunming University, Kunming, China; ^2^Department of Creative Arts Therapy, Jeonju University, Jeonju, Republic of Korea

**Keywords:** art therapy, Chinese students, South Korea, acculturative stress, academic stress, study abroad

## Abstract

**Background:**

Research has shown that international students, specifically Chinese graduate students in South Korea, are vulnerable to stress and depression because of various factors. These include environmental changes, economic constraints, interpersonal difficulties, discrimination, and cultural conflict.

**Objective:**

This study investigates the effectiveness of group art therapy in reducing acculturative stress and academic stress among Chinese graduate students in South Korea.

**Method:**

Thirty participants were recruited and randomly assigned to the experimental (*n* = 15) and control groups (*n* = 15). The experimental group received eight 120-min sessions of group art therapy. Both groups were tested for acculturative stress (perceived discrimination, homesickness, perceived hate, fear, stress due to change/culture shock) and academic stress (schoolwork stress, future stress, social stress, living environment stress) before and after the art therapy intervention. Results were compared using the Mann–Whitney U test and the Wilcoxon signed-rank test.

**Results:**

The results showed that there was a significantly greater reduction in acculturative and academic stress in the experimental than the control group.

**Conclusion:**

Group art therapy can provide psychological and emotional support to international students studying abroad.

## 1. Introduction

With the rapid development of globalization, exchanges between countries are becoming increasingly active. Since the establishment of diplomatic relations between China and South Korea in 1992, there has been continuous communication between the nations in politics, economics, culture, education, and other fields (Luo, 2022; Yang, 2022). According to a survey conducted by the Korean Educational Statistics Service (2022), there are 166,892 foreign students residing in South Korea, of which 67,439 (40%) are Chinese students.

However, Chinese students who come to South Korea to obtain a degree often face difficulties in adapting to university life due to their insufficient language ability, differences in beliefs and mindset, and lack of adequate assistance in academic and daily life (Zhao and Lee, 2018; Qiao, 2020; Song et al., 2020). The representative challenge faced by Chinese graduate students in South Korea is communication difficulty due to language barriers. In addition to studying the academic subjects, they also have to learn the Korean language and culture, which adds to their academic burden.

The depression, anxiety, and mental stress experienced by students in dealing with school life and academic requirements can be defined as a state of psychological maladjustment (Kim et al., 2014; Shin et al., 2017). When academic pressure exceeds personal abilities, it causes psychological strain and social isolation, leading to maladjustment and ultimately depression (Rice et al., 2016; Zhao, 2016). Indeed, Chinese graduate students who leave their homeland for South Korea with aspirations for academic achievement experience various difficulties in the adaptation process. In leaving the familiar cultural circle and during the early stages of their study in South Korea, these students generally experience emotional and physical stress, academic maladjustment, as well as environmental, linguistic, and dietary discomfort (Hu, 2011; Hoang, 2021). Economic burdens, difficulties in interpersonal relationships, family expectations, and complexities in self-identity formation also increase the cultural adaptation pressure on international students, leading to psychological stress (Xu, 2021). Thus, Chinese graduate students in South Korea face psychological burdens and inconveniences in daily life due to environmental and role changes, identity confusion, discrimination, and cultural conflicts (Park et al., 2010; Lee and Yoo, 2022).

Culture adaptation pressure can cause negative impacts on mental health, day-to-day functioning, and overall well-being and is accompanied by the following: physical, psychological, and social confusion; anxiety; depression; alienation; serious physical symptoms; and identity confusion. A study conducted with Chinese students visiting school counseling centers showed that 50% of them were in a state of depression, mainly due to cultural adaptation pressure and academic pressure (Zhao, 2016). In addition, various research collectively indicate that there is a meaningful connection between the cultural adaptation difficulties of Chinese students in South Korea and the academic pressure they feel, including the following: empirical surveys on Chinese students in South Korea (Park, 2009; Yang, 2013; Kang and Ko, 2019); qualitative studies on Chinese graduate students’ adaptation to university life in South Korea (Park et al., 2010; Lee, 2012; Lee and Kim, 2015; Kim and Yoon, 2017); studies on the relationship between cultural adaptation pressure, academic pressure, and adaptation to university life as a single variable (Kim, 2007; Kim Y. K. 2010; Park et al., 2010). Foreign students who experience cultural adaptation and academic pressure tend to develop mental health problems such as anxiety or depression (Koung, 2010; Zhao et al., 2023). Moreover, like native Korean students, upon graduation, Chinese students will also be expected to play their roles as productive and responsible members of the society they settle down into. Therefore, their psychological and emotional well-being is not only important for themselves but also for the entire society.

Art therapy can be used as a nonverbal method to assist language-deficient international students from China to adapt to the Korean culture and achieve academic success. By offering a space for free exploration and creative work, art therapy can provide the following: facilitate the expression of things that are difficult or impossible to articulate in words; evoke numerous defense mechanisms; induce responses that may be ambiguous or confusing (Rubin, 1984; Greenberg, 2017). Thus, art therapy and art making can serve as effective means of emotional expression for individuals with emotional or psychological difficulties, including those who have difficulty expressing their emotions directly (Wadeson, 1987; Malchiodi, 2005). By stimulating internal group interactions, art therapy delivers a platform for people to discuss life issues and improve their interpersonal relationship skills (Park, 2020), then reducing academic stress and deepening life satisfaction (Kim, 2022). Cho (2022) showed that painting-centered group art therapy can help foreign students enhance their self-awareness and expression, increase intimacy within the group, reduce stress in the community, and improve quality of life. These previous studies collectively suggest that group art therapy has a positive impact on reducing stress. However, no researcher thus far has examined group art therapy application to alleviate cultural adaptation and academic stress among Chinese graduate students in South Korea.

This evokes the need for research attempting to verify whether group art therapy can reduce the acculturative and academic stress commonly faced by Chinese graduate students in South Korea. Accordingly, in this study, we hoped to propose a group art therapy intervention that helps to alleviate the physical and psychological stress experienced by these students, specifically through encouraging them to engage in a therapeutic creative activity in a supportive environment. We expected that this could help them cope with the acculturative and academic stress that they experienced. We also conducted this study in the hopes to lay the foundation for the development of effective group art therapy programs applicable to real clinical settings for Chinese graduate students in South Korea. To confirm the effectiveness of group art therapy on acculturative stress and academic stress in this population, the following research questions were tested.

Research Question 1: Does group art therapy affect the acculturative stress of Chinese graduate students in South Korea?

Research Question 2: Does group art therapy affect the academic stress of Chinese graduate students in South Korea?

## 2. Research method

To recruit participants for the experiment, the background and purpose of the study were posted on a network chat software (WeChat) used by Chinese graduate students in South Korea. The recruitment period for participants was from March 20 to April 20, 2022, and applications from voluntary participants were received *via* the researcher’s WeChat or email. After the announcement ended, 30 applicants who met the recruitment criteria were selected for the study. The criteria for participation included enrollment in a degree program in Korea, being raised and born in mainland China, experiencing acculturative and academic stress, and the ability to participate in eight face-to-face sessions of group art therapy. The recruited participants were randomly divided into an experimental group of 15 and a control group of 15.

A survey questionnaire was distributed to investigate participants’ general demographic characteristics (Table 1). Results of an analysis of these characteristics showed that, regarding biological sex, there were 10 men (66.7%) and 5 women (33.3%) and 12 men (80.0%) and 3 women (20.0%) in the experimental and control groups, respectively. Regarding residency in South Korea, in the experimental group, 4 participants (26.7%) had stayed for less than 12 months and 11 participants (73.3%) had stayed for more than 12 months in the country; in the control group, the corresponding numbers were 6 (40.0%) and 9 participants (60.0%), respectively. Thus, the control group had a closer balance of newer and older residents.

**Table 1:** Demographic characteristics of participants.

Domain	Area	EG (*n* = 15)		CG (*n* = 15)		Total	
		*n*	%	*n*	%	*n*	%
Sex	Male	10	66.7	12	80.0	22	73.3
	Female	5	33.3	3	20.0	8	26.7
Residency in South Korea (months)	< 12	4	26.7	6	40.0	10	33.3
	≥ 12	11	73.3	9	60.0	20	66.7
TOPIK level	Under 2	12	80.0	10	66.7	22	73.3
	Over 3	3	20.0	5	33.3	8	26.7
Major	Arts	10	66.7	2	13.3	12	40.0
	Natural Sciences	5	33.3	5	33.4	10	33.3
	Humanities and Social Sciences	0	0.0	8	53.3	8	26.7
Degree	Bachelor and Master’s degree	7	46.7	4	26.7	11	36.7
	Doctorate	8	53.3	11	73.3	19	63.3

EG, experimental group; CG, control group.

In terms of TOPIK (Test of Proficiency in Korean) level, in the experimental group, 12 participants (80.0%) had a level below 2 and 3 participants (20.0%) had a level of 3 or higher; among the 12 students with a level below 2, 10 students (66.7%) did not have a TOPIK level, which is a relatively high percentage. In the control group, 10 participants (66.7%) had a level below 2—9 of which (60.0%) did not have a TOPIK level—and 5 participants (33.3%) had a level of 3 or higher. In terms of academic degree, there were 7 master’s degree students (46.7%) and 8 doctoral students (53.3%) in the experimental group, and 4 master’s degree students (26.7%) and 11 doctoral students (73.3%) in the control group. Thus, more than half of the participants in both the experimental and control groups were doctoral students.

### 2.1. Measurement instrument

The Acculturative Stress for International Students (ASIS) and Academic stress scales were administered individually to participants 1 week before the start of the project and again upon completion of the program.

#### 2.1.1. Acculturative stress scale

Originally designed by Sandhu and Asrabadi (1994) and modified by Lee (1995) to evaluate the cultural adaptation stress of international students in South Korea, this study used the modified version of the Acculturative Stress for International Students (ASIS) scale to assess acculturative stress. The modified self-assessed 24-item scale (“cultural adaptation stress scale”) measures the following seven sub-factors of acculturative stress: “perceived discrimination” (9 items), “homesickness” (3 items), “perceived hate/rejection” (5 items), “fear” (4 items), “stress due to change/culture shock” (3 items). Each item is self-assessed using a 5-point Likert scale, with higher scores indicating higher levels of stress. The results of the reliability test of the acculturative stress scale are shown in Table 2.

**Table 2:** Sub-factors and reliability of the cultural adaptation stress scale.

Sub-factors	Item identifier	Number of items	Cronbach’s α
Perceived discrimination	3, 7, 8, 10, 12, 14, 17, 19, 21	9	0.842
Homesickness	1, 5, 24	3	0.688
Perceived hate	4, 11, 15, 18, 23	5	0.745
Fear	6, 13, 20, 22	4	0.787
Stress due to change/culture shock	2, 9, 16	3	0.758
All	24		0.923

In this study, the overall reliability of the acculturative stress scale was 0.923, and that of its sub-factors were 0.842 (“perceived discrimination”), 0.688 (“homesickness”), 0.745 (“perceived hate/rejection”), 0.787 (“fear”) and 0.758 (“stress due to change/culture shock”).

#### 2.1.2. Academic stress scale

In previous studies, Oh and Cheon (1994) developed an academic stress questionnaire, Chon et al. (2000) developed a life stress questionnaire, and Kim (2018) supplemented both these questionnaires with language suitable for college students in order to create a modified scale to assess academic stress. This scale created by Kim (2018) was then translated into Chinese by Li (2019), and Li’s (2019) scale was the one used in the current study for assessing academic stress.

This self-assessed 23-item scale comprises four sub-factors, as described herein: “schoolwork stress” (9 items), “future stress” (5 items), “social stress” (5 items), and “living environment stress” (4 items). Each question was answered using a 5-point Likert scale, with higher scores indicating greater academic stress.

Reliability checks were conducted on the collected data, and results are shown in Table 3. In this study, the overall reliability of this scale was 0.879, and the reliability of each sub-factor was as follows: 0.655 (“schoolwork stress”), 0.752 (“future stress”), 0.766 (“social stress”), and 0.792 (“living environment stress”). All questions showed an appropriate level of reliability.

**Table 3:** Sub-factors and reliability of academic stress scale.

Sub-factors	Item identifier	Number of items	Cronbach’s α
Schoolwork stress	1, 2, 3, 4, 5, 6, 7, 8, 9	9	0.655
Future stress	10, 11, 12, 13, 14	5	0.752
Social stress	15, 16, 17, 18, 19	5	0.766
Living environment stress	20, 21, 22, 23	4	0.792
All	23		0.879

## 3. The Group Art Therapy Project

### 3.1. Design model of the Group Art Therapy Project

To determine the impact of group art therapy on cultural adaptation stress among Chinese graduate students in South Korea, this study conducted a pretest on the experimental and control groups. Only the experimental group received group art therapy. Subsequent tests were conducted on both the experimental and control groups, as shown in Table 4.

**Table 4:** Study design model.

Group	Pretest	Experimental treatment	Posttest
EG (*n* = 15)	Q1	X	Q2
CG (*n* = 15)	Q3	–	Q4

EG, experimental group; CG, control group; Q1 and Q3, pretest (acculturative stress, academic stress); X, eight sessions of group art therapy; –, control group with no treatment; Q2 and Q4, posttest (acculturative stress, academic stress).

The Group Art Therapy Project was conducted from June 4 to June 28, 2022, on Tuesdays and Saturdays at 2 p.m. for a total of 8 sessions. Considering translation time and group size, with the agreement of group members, each session lasted approximately 120 min. To ensure the safety of participants, the group art therapy was conducted in a classroom in a university located in South Korea.

In selecting the art therapist for the Project, a professional with a master’s degree in Art Therapy and more than 8 years of experience in group therapy was chosen. An expert who has provided art therapy for multicultural clients for several years also verified the project. During the project sessions, a Chinese translator was present to support the communication between the participants and the art therapist. With the consent of the participants, the researchers also participated as observers.

### 3.2. Project content

This study aimed to demonstrate the effectiveness of group art therapy in reducing cultural adaptation stress and academic stress among Chinese graduate students in South Korea. Thus, the therapy focused on stabilizing emotions, expressing emotions and thoughts, and reducing stress related to cultural adaptation and academic pressure. Additionally, the therapy aimed to help students understand and develop themselves through art. The process of the Group Art Therapy Project is briefly presented in Table 5.

**Table 5:** Process of group art therapy.

Phase	Session #	Theme	Purpose
Initial period	1	Self-introduction activities	– Self-introduction and formation of group cohesion.
	2	The culture I have lived in	– Awareness of the nature of culture. – Share experiences with group members and receive emotional support.
Intermediate period	3	Difficulties I encountered	– Expression of conflict between culture and personal nature. – Share the difficulties in the process of cultural adaptation.
	4	Five facial expressions	– Recognize feelings and express them.
	5	The wall in front of me	– Emotional expression and empathic experiences of academic stress. – Improve problem-solving skills to relieve academic stress.
	6	The miracle question of Aladdin’s lamp	– Exploration of inner desires and their manifestation. – Share experiences with others about desire for support and receiving support.
Final period	7	The card that represents me	– Pursue life and self-image expression. – Internal resources to explore and motivate goals.
	8	A gift box for me	– Inner positive self-image.

**Figure 1:**
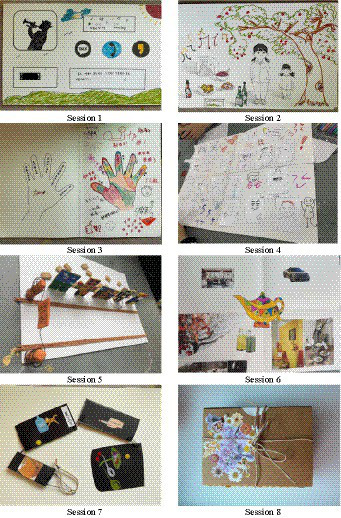
Representative art products of each session of the Group Art Therapy Project.

The Project consisted of three phases within total of eight sessions, namely the initial (session 1 and 2), intermediate (session 3–6), and final periods (session 7–8). In the initial phase, a sense of closeness was formed between participants, generating interest in art therapy and guiding them towards self-expression. In the intermediate phase, participants explored their negative emotions, cultural adaptation stress, and academic stress, showing self-awareness and suppressed emotions. Additionally, through collective resonance and support, the recognition of external and internal resources was encouraged. In the final phase, participants formulated concrete future plans through self-identification and acceptance, focusing on the formation of a positive self-image and a strategy to overcome stressful situations in the future. Examples of the products of art produced in each session are presented in Figure 1.

In each stage of the Group Art Therapy Project, during the introduction step, participants greeted each other and conversed about their current mood. In the execution step, the background, purpose, and sequence of the program were explained. At this point, the therapist first demonstrated or explained the assignment. If additional assistance was required, the researchers played the role of assistant therapists. After the program ended, participants introduced their work and shared their thoughts and feelings on each other’s combined work.

### 3.3. Data analysis

SPSS version 26.0 was used in all analyses. First, we verified the reliability of both scales used in this study through internal consistency reliability (Cronbach’s α). Second, to check the homogeneity of the experimental and control groups before program implementation, a Mann–Whitney U test was performed. Third, to investigate differences in the scores of the two groups regarding acculturative stress and academic stress between pretest and posttest, a Wilcoxon signed-rank test was performed.

### 3.4. Ethical consideration

The therapy activities and data collection procedures of this study were approved by the relevant institution review board (JJ IRB-220421-HR-2022-0407). This paper is a modified version of part of a doctoral dissertation (Yin, 2023).

## 4. Results

### 4.1. Changes in acculturative stress

#### 4.1.1. Homogeneity verification for adaptation stress

We investigated the homogeneity of cultural adaptation stress scores between groups before project implementation using Mann–Whitney U test (Table 6). At pretest, the difference between groups for the total score of cultural adaptation stress was non-significant (*p* = 1.000), and the differences between groups in the scores for all sub-factors were also non-significant, as follows: “perceived discrimination” (*p* = 0.917), “homesickness” (*p* = 0.131), “perceived hate/rejection” (*p* = 0.148), “fear” (*p* = 0.061), and “stress due to change/culture shock” (*p* = 0.645).

**Table 6:** Homogeneity verification of acculturative stress between groups.

Lower factor	EG (*n* = 15)		CG (*n* = 15)		*Z*
	*M*	SD	*M*	SD	
Perceived discrimination	3.27	0.630	3.21	1.152	−0.104
Homesickness	4.09	0.761	3.60	0.910	−1.510
Perceived hate/rejection	3.60	0.605	3.20	0.855	−1.480
Fear	3.63	0.870	2.93	1.193	−1.875
Stress due to change/culture shock	3.47	0.915	3.20	1.265	−0.605
All	3.53	0.592	3.21	1.010	−0.913

EG, experimental group; CG, control group.

The mean scores for cultural adaptation stress and its sub-factors were higher in the experimental group than in the control group, but these differences were not statistically significant (*p* > 0.05). These results suggested homogeneity between groups for cultural adaptation stress.

#### 4.1.2. Pretest and posttest score differences for cultural adaptation stress

To assess the effect of group art therapy on cultural adaptation stress, we compared differences between pretest and posttest scores for both groups using Wilcoxon signed-rank test. We also compared the posttest scores for cultural adaptation stress between groups (Table 7).

**Table 7:** Differences in pretest and posttest scores for acculturative stress in the experimental group.

Lower factor	Pre-post		Ex-post		*Z*
	*M*	SD	*M*	SD	
Perceived discrimination	3.27	0.630	1.58	0.455	−3.412**
Homesickness	4.09	0.761	1.91	0.980	−3.417**
Perceived hate/rejection	3.60	0.605	1.80	0.646	−3.413**
Fear	3.63	0.870	1.65	0.632	−3.417**
Stress due to change/culture shock	3.47	0.915	1.73	0.594	−3.302**
All	3.53	0.592	1.70	0.558	−3.408**

**p < 0.01.

In the experimental group, the total score for cultural adaptation stress decreased significantly from pretest to posttest (pretest, 3.53; posttest, 1.70; *p* < 0.01). The decrease was similarly significant for all sub-factors, namely “perceived discrimination” (pretest, 3.27; posttest, 1.58; p < 0.01), “homesickness” (pretest, 4.09; posttest, 1.91; *p* < 0.01), “perceived hate/rejection” (pretest, 3.60; posttest, 1.80; p < 0.01), “fear” (pretest, 3.63; posttest, 1.65; *p* < 0.01), and “stress due to change/culture shock” (pretest, 3.47; posttest, 1.73; *p* < 0.01; Table 8).

**Table 8:** Difference in pretest and posttest scores for acculturative stress in the control group.

Lower factor	Pre-post		Ex-post		*Z*
	*M*	SD	*M*	SD	
Perceived discrimination	3.21	1,152	3.48	1.293	−0.629
Homesickness	3.60	0.910	4.00	1.069	−1.260
Perceived hate/rejection	3.20	0.855	3.63	1.146	−1.053
Fear	2.93	1.193	3.55	1.347	−1.386
Stress due to change/culture shock	3.20	1.265	3.58	1.324	−1.018
All	3.21	1.010	3.60	1.209	−1.022

In the control group, the total score for cultural adaptation stress increased non-significantly from pretest to posttest (pretest, 3.21; posttest, 3.60; *p* > 0.05). The increase was similarly non-significant for all sub-factors of “perceived discrimination” (pretest, 3.21; posttest, 3.48; *p* > 0.05), “homesickness” (pretest, 3.60; posttest, 4.00; *p* > 0.05), “perceived hate/rejection” (pretest, 3.20; posttest, 3.63; *p* > 0.05), “fear” (pretest, 2.93; posttest, 3.55; *p* > 0.05), and “stress due to change/culture shock” (pretest, 3.20; posttest, 3.58; *p* > 0.05).

Thus, the Group Art Therapy Project significantly reduced cultural adaptation stress in the experimental group, but not in the control group. This suggests the positive impact of the therapy project on reducing cultural adaptation stress among Chinese graduate students in South Korea.

Upon comparing posttest scores for cultural adaptation stress between groups, we observed that the scores for all sub-factors and total score were significantly lower in the experimental than in the control group (*p* < 0.01). This once more suggests that the Group Art Therapy Project had a more positive impact on the total score and scores for all sub-factors of cultural adaptation stress in the experimental than in the control group, and the effect was significant.

### 4.2. Changes in academic stress

#### 4.2.1. Homogeneity verification for academic stress

Again, we tested the homogeneity of academic stress scores in the groups before project implementation. At pretest, the difference between groups for the total score of academic stress was non-significant (*p* = 1.000), and the differences between groups in the scores for the sub-factors of academic stress were also non-significant, as described herein: “schoolwork stress” (*p* = 0.454), “future stress” (*p* = 0.546), “social stress” (*p* = 0.646), “living environment stress” (*p* = 0.573; Table 9).

**Table 9:** Verification of homogeneity of academic stress between groups.

Lower factor	EG (*n* = 15)		CG (*n* = 15)		*Z*
	*M*	SD	*M*	SD	
Schoolwork stress	4.25	0.379	3.96	0.836	−0.749
Future stress	4.07	0.766	3.75	1.070	−0.604
Social stress	3.19	0.860	2.93	1.267	−0.459
Living environment stress	3.58	1.063	3.40	1.113	−0.564
All	3.86	0.547	3.59	0.899	−1.100

EG, experimental group; CG, control group.

The mean scores for academic stress and its sub-factors were higher in the experimental group than in the control group, albeit the difference between groups was non-significant (*p* > 0.05). These results suggested homogeneity between groups for academic stress.

#### 4.2.2. Pretest and posttest score differences in academic stress

Wilcoxon signed-rank test was used to verify differences between pretest and posttest scores for academic stress in both groups. We also compared posttest scores for academic stress between groups (Table 10).

**Table 10:** Differences in pretest and posttest scores for academic stress in the experimental group.

Lower factor	Pre-post		Ex-post		*Z*
	*M*	SD	*M*	SD	
Schoolwork stress	4.25	0.379	2.28	1.123	−3.237**
Future stress	4.07	0.766	2.07	0.827	−3.326**
Social stress	3.19	0.860	1.80	0.986	−3.079**
Living environment stress	3.58	1.063	1.83	1.068	−3.953**
All	3.86	0.547	2.05	0.963	−3.295**

**p < 0.01.

In the experimental group, the total score for academic stress decreased significantly from pretest to posttest (pretest, 3.86; posttest 2.05; *p* < 0.01). The decrease was also significant for all sub-factors of academic stress, as follows: “schoolwork stress” (pretest, 4.25; posttest, 2.28; *p* < 0.01), “future stress” (pretest, 4.07; posttest, 2.07; *p* < 0.01), “social stress” (pretest, 3.19; posttest, 1.80; *p* < 0.01), “living environment stress” (pretest, 3.58; posttest, 1.83; *p* < 0.01).

In the control group (Table 11), the total score for among the academic stress decreased significantly from pretest to posttest (pretest, 3.59; posttest 3.69; *p* < 0.01). the changes in the scores for the sub-factors of academic stress from pretest to posttest were all non-significant, and as follows: increased for “schoolwork stress” (pretest, 3.96; posttest, 4.04; *p* > 0.05), “social stress” (pretest, 2.93; posttest, 3.17; *p* > 0.05), and “living environment stress” (pretest, 3.40; posttest, 3.67; *p* > 0.05); decreased for “future stress” (pretest, 3.75; posttest, 3.51; *p* > 0.05).

**Table 11:** Pretest and posttest scores for academic stress in the control group.

Lower factor	Pretest		Posttest		*Z*
	*M*	SD	*M*	SD	
Schoolwork stress	3.96	0.836	4.04	0.937	−0.221
Future stress	3.75	1.070	3.51	1.202	−0.833
Social stress	2.93	1.269	3.17	1.278	−0.315
Living environment stress	3.40	1.113	3.67	1.227	−1.105
All	3.59	0.899	3.67	1.049	−0.142

Thus, the experimental group experienced a significant decrease in academic stress, while the control group experienced a non-significant change for this variable. These findings suggest that group art therapy has a positive effect on reducing academic stress in Chinese graduate students in South Korea.

Upon comparing the post-hoc tests for academic stress among the experimental and control groups, the results showed that the scores for the total scale and all sub-factors of academic stress were significantly lower in the experimental group compared with the control group (*p* < 0.01). In other words, the Group Art Therapy Project had more positive effects on the total scale and all sub-factors of academic stress in the experimental group compared with the control group, with significant results.

## 5. Discussion

### 5.1. Changes in cultural adaptation stress

Our results for the comparison of pretest and posttest scores for cultural adaptation stress in both groups showed a significant reduction in the experimental group. Thus, group art therapy showed positive effects on overall cultural adaptation stress and its underlying factors in the study sample. The findings also demonstrated that the scores for the total scale and sub-factors of cultural adaptation stress at posttest were significantly lower in the experimental than in the control group.

These results are consistent with those of previous studies conducted with Korean and Chinese international students by Cho (2022), Park (2013), Hu (2011), and Lee (2008), and with a study on female immigrant marriage by Kwon (2009). Therefore, the evidence suggests that expressing personal emotions and experiences through artistic media for cultural adaptation stress should ideally not be a solitary activity. This is because collective psychological support experiences have a more positive impact on relieving cultural adaptation stress. From this perspective, sharing personal experiences, expressing feelings, finding empathy, and understanding from kindred souls, collectively discussing solutions to challenges, and listening to others’ stories may have given the experimental group participants a sense of comfort and relieved their cultural adaptation stress.

### 5.2. Changes in academic stress

Our findings showed that there was a significant reduction in overall academic stress and its sub-factors in the experimental group. They also indicated that the experimental group had significantly lower scores for overall academic stress and its sub-factors at posttest than the control group.

These findings are consistent with those of the studies by Kim (2022), which was focused on the implementation of non-face-to-face collective art therapy with college students, and Wang (2022), which delved into the effects of fusion art therapy programs for Korean and Chinese international students. Both cited studies showed the positive effects of art therapy in reducing academic stress. These results suggest that the expression and dissolving of negative emotions induces psychological relaxation, resulting in reduced academic stress (Kim H. J., 2010; Lee, 2012; Kim, 2017).

### 5.3. Limitations and future directions

This study has the following limitations. First, the study focused on Chinese graduate students studying in a university in A city, South Korea, with limited selection criteria and sample size. Therefore, it is difficult to generalize the findings to all Chinese graduate students in South Korea. Participants were selected based on voluntary participation, which limited the possibility of screening. Therefore, it is suggested for future research to expand the scope of the study, such as by increasing the number of participants and selecting them based on screening to identify those facing challenges with cultural adaptation and academic stress.

Second, eight sessions of group art therapy were conducted over 4 weeks. The short duration of the intervention hinders, once more, our ability to generalize the findings. Therefore, future research should investigate the effectiveness of long-term group art therapy programs.

Third, the study did not conduct follow-up evaluations to examine the sustained effects of the intervention. Therefore, it is suggested that future research measure and validate changes in the levels of cultural adaptation stress and academic stress among Chinese graduate students in South Korea through follow-up evaluations after the completion of the group art therapy program.

This study shows that providing group art therapy to Chinese graduate international students in South Korea, who may have difficulties in adapting to the new culture and face academic stress, can help alleviate cultural adaptation stress and academic stress. Moreover, it can be used to help international students optimize their potential to make academic achievements in a foreign environment. This study contributes to the exploration of psychological and emotional support solutions for Chinese graduate international students studying in a foreign country, particularly South Korea. It is hoped that this study will serve as a useful reference for various types of therapeutic programs for such students.

## Data availability statement

The raw data supporting the conclusions of this article will be made available by the authors, without undue reservation.

## Ethics statement

The studies involving human participants were reviewed and approved by IRB at Jeonju University JJ IRB-220421-HR-2022-0407. The patients/participants provided their written informed consent to participate in this study.

## Author contributions

YY, as the first author, completed the initial draft of the article along with the experiments, data collection, and data analysis. KK as the corresponding author, provided feedback, revised, and edited the first draft of the article. All authors contributed to the article and approved the submitted version.

## Conflict of interest

The authors declare that the research was conducted in the absence of any commercial or financial relationships that could be construed as a potential conflict of interest.

## Publisher’s note

All claims expressed in this article are solely those of the authors and do not necessarily represent those of their affiliated organizations, or those of the publisher, the editors and the reviewers. Any product that may be evaluated in this article, or claim that may be made by its manufacturer, is not guaranteed or endorsed by the publisher.
